# HIV serostatus disclosure and associated factors among HIV positive pregnant and lactating women at Nekemte public health facilities, western Ethiopia

**DOI:** 10.1371/journal.pone.0248278

**Published:** 2021-03-19

**Authors:** Tadesse Tolossa, Bizuneh Wakuma, Merga Besho, Diriba Mulisa, Ginenus Fekadu, Lami Bayisa, Reta Tsegaye

**Affiliations:** 1 Department of Public Health, Institutes of Health Sciences, Wollega University, Nekemte, Ethiopia; 2 School of Nursing and Midwifery, Institutes of Health Sciences, Wollega University, Nekemte, Ethiopia; 3 School of Pharmacy, Institutes of Health Sciences, Wollega University, Nekemte, Ethiopia; Medical University of Warsaw, POLAND

## Abstract

**Background:**

Disclosure of Human Immune Virus (HIV) serostatus by pregnant and lactating women is crucial for the successful prevention of mother to child transmission of HIV/AIDS. However, little has been studied regarding the prevalence and factors associated with HIV status disclosure among HIV positive pregnant and lactating women in Ethiopia.

**Methods:**

An institution-based cross-sectional study was conducted in the Nekemte Public Health facilities among 380 pregnant and lactating women enrolled in universal antiretroviral therapy (ART) treatment from January 2015-December, 2019. The data were collected by using a checklist, developed from Prevention of Mother to Child Transmission (PMTCT) logbook, ART intake forms, and medical cards of the patients. Epidata version 3.2 was used for data entry, and then the data were exported to STATA version 14 for further analysis. The binary logistic regression model was employed to determine factors associated with the disclosure status among HIV positive pregnant and lactating women. Adjusted Odds Ratio (AOR) with 95% confidence intervals was computed and statistical significance was declared when it is significant at a 5% level (p-value < 0.05).

**Results:**

A total of 380 women have participated in the study. Two hundred seventy-six (73.4%) of women had disclosed their HIV status to at least one individual. The study found living in urban (OR = 1.83, 95% CI: 1.04, 3.20), married women (OR = 4.16, 95% CI: 1.87, 9.24), higher educational status (OR = 2.35, 95% CI: 1.31, 5.51), positive HIV status of partner (OR = 2.35, 95%CI: 1.17, 4.70), and being multipara (OR = 4.94, 95% CI: 2.29, 10.66) were independent determinants of HIV status disclosure.

**Conclusions:**

HIV status disclosure among pregnant and lactating women in the study area was sub-optimal. Empowering women through education, encouraging partners for HIV testing, and enhancing active male involvement in HIV treatment and control programs should get due attention.

## Introduction

HIV disclosure is defined as the act of telling to a third party that someone is seropositive. These acts can either be ‘voluntary’ (by the initiative of a person living with HIV) or ‘involuntary’ (when the seropositivity status is disclosed to the third party without the consent of the person living with HIV) [1]. Disclosure serostatus has often been associated with increased social support, facilitate engagement in care, and treatment. Disclosure to sexual partners could also increase rates of HIV testing and reduce transmission risk behaviors [2].

Currently, an increasing number of pregnant women are being tested for HIV worldwide as efforts to decrease perinatal HIV transmission [3]. HIV-positive test in the context of antenatal care typically differ in a way that pregnant women test positive when they may feel healthy, rather than through voluntary counseling and testing or provider-initiated testing [4].

Disclosure among pregnant and lactating women has unique implications. It is proved in increasing the effectiveness of the prevention of mother-to-child transmission (PMTCT) service and a significant effect on decreasing sexual transmission of HIV. Disclosure of serostatus to the spouse was significantly associated with exclusive breastfeeding practices and antiretroviral uptake [5]. HIV status disclosure to partners by pregnant and lactating women was also associated with increased spousal support [6].

The rate of serostatus disclosure among pregnant and lactating women vary across low-income countries and it ranges from 16.7% to 86%, with the lowest rates (16.7% to 32%) of being among pregnant women in Sub-Saharan Africa (SSA) [7]. HIV status disclosure remains a challenge for many women in developing countries as it is impeded by adverse outcomes of disclosure such as extreme as physical assault, rejection, stigma, discrimination, and violence [6]. In SSA, fear of HIV serostatus disclosure has been among the common barriers to PMTCT program uptake [7].

Studies have found women’s social and economic circumstances are central determinants of the disclosure. The decisions of pregnant women to disclose their seropositivity status are influenced by social norms, family dynamics, age group, type of social relationships, income, educational level, health access and quality, levels of perceived stigma, parity, history of domestic violence and financial independence, quality of housing and residing [8, 9].

In Ethiopia, the level of HIV serostatus disclosure among general population is below what the government intends to have in the country. Literature shows the prevalence of disclosure is not only at its lower level, but it is also highly variable across different parts of Ethiopia. In Ethiopia, the pooled prevalence of disclosure in the general population was 73% [9]. A study conducted in Addis Ababa, Ethiopia showed the serostatus disclosure of HIV pregnant women to their partners were 73% [10]. HIV status disclosure among pregnant and lactating women was 85.7% in Hawasa [11], 89.7 in Northwest Ethiopia [12], and 63.8% in Mekelle town [13].

In general providing support to encourage women in safely disclosing their status is needed. The purpose of this study was to identify factors associated with HIV disclosure status among pregnant and lactating women who test HIV positive. This finding could also be helpful in developing effective HIV prevention and control strategies in the country.

## Methods

### Methods study area and study design

An Institution-based cross-sectional study was conducted in the Nekemte Public Health facilities (Nekemte specialized Hospital and Nekemte Health Center) among pregnant and lactating women enrolled to universal ART treatment from January 2015-December, 2019. Nekemte Specialized Hospital and Nekemte Health center are found in Nekemte town, the capital city of East Wollega Zone. Nekemte is located 328 kilometer from Addis Ababa to the western part of the country. Data were extracted from client cards from March 15–30, 2019.

### Populations

All pregnant and lactating women enrolled in universal ART treatment found in Nekemte public Health facilities were source populations. Study populations were all pregnant and lactating women enrolled to universal ART treatment at Nekemte Specialized Hospital and Nekemte Health center from January 2015-December, 2019.

### Eligibility criteria and participants

All pregnant and lactating women initiated ART at PMTCT clinic of Nekemte Specialized Hospital and Nekemte Health center from January 2015-December, 2019 were included in the study. A women who were transferred out to other facilities during the study period, or whose intake form was not complete was excluded from this study. All eligible women (n = 380) during the specified time period were included in the study.

### Outcome measurement

The outcome variable was HIV positive pregnant and lactating women disclosure status (Yes/No). Disclosure status was recorded as “Yes” if the women disclosed their HIV status to one of the followings (sexual partner, husband, spouse, sister and brother, relatives) and “No” if the women do not disclose their HIV status to one of the followings (partner, husband, spouse, sister and brother, relatives).

Independent variables were socio-demographic factors of women (age, residence, marital status, employment status, educational level, and religion), maternal related factors (partner HIV status, the status of the women at the initiation of ART, previous History of HIV, and the number of pregnancy on the initiation of ART), baseline clinical and laboratory factors (baseline CD4 count, baseline WHO disease stage, baseline Hgb level, baseline OI’s, baseline functional status, comorbidity, baseline TB Screening, and recent OI’s) and baseline regimen related factors (types of baseline regimen, recent ART adherence, recent drug side effect, and baseline CPT).

Functional status was categorized as working (able to perform usual work in or out of the house), ambulatory (able to perform activities of daily living), and bedridden (not able to perform activities of daily living) [14]. The level of ART adherence level was recorded as good adherence (if the missed dose is ≤ 2 doses of 30 prescribed doses or < 3 doses of 60 prescribed doses as documented by ART physician), fair adherence level (if the missed dose is between 3–5 doses of 30 prescribed doses or 3–9 doses of 60 prescribed doses as documented by ART physician), poor adherence level (if the missed dose is ≥ 6 doses of 30 prescribed doses or >9 doses of 60 prescribed doses as documented by ART physician [14]. The previous history of HIV was categorized as “No” if women newly enrolled to PMTCT clinic and had no previous history of HIV, and “yes” if women had the previous history of HIV and on ART follow-up before entry to PMTCT.

### Data collection tools, procedure and quality assurance

The data were collected by using a checklist, developed from the PMTCT logbook, ART intake forms and medical cards of the patients. The checklist consists of women socio-demographic data, maternal related data, baseline clinical and laboratory data of the women, and regimen related data.

Data were collected from the cards of pregnant and lactating women who under universal; ART treatment from January 2015 to December 2019. The outcome variable, disclosure status was identified as “Yes/No” by reviewing PMTCT registration logbook recorded by health professionals. One trained nurse working at the PMTCT clinic for each health institution was recruited for data collection. The one-day training was given for data collectors by the principal investigator. A pre-test was conducted on 5% of patient records before the actual data collection to assess the clarity of the checklist, and variable present on the logbook at Nekemte Specialized hospital. Accordingly, the possible amendment was made (variables such as baseline viral load, and pre ART counseling were removed from the checklist).

### Data management and analysis

Epi data version 3.2 was used for data entry, and then the data were exported to STATA version 14 for further analysis. Descriptive statistics, like frequencies, percentages, mean and standard deviation were computed. Before analysis, data were cleaned and edited by using simple frequencies and cross-tabulation. To be suitable for analysis, the re-categorization of categorical variables and categorization of continuous variables was done. The assumption of the logistic regression model was checked by the chi-square test. The binary logistic regression model was employed to determine factors associated with disclosure status among HIV positive pregnant and lactating women. Factors that were associated with the outcome variable at 25% (P-value ≤0.25) significant level in the bivariable logistic regression analysis were included in the multivariable logistic regression analysis by using a forward stepwise selection process. Then crude and adjusted odds ratio together with their corresponding 95% confidence intervals were presented in the final multivariable logistic regression table. AOR with 95% confidence intervals was computed and statistical significance was declared when it is significant at a 5% level (p-value < 0.05).

### Ethical approval

Ethical approval was obtained from the ethical review committee of Wollega University, Institute of Health and support letter was written to each health facilities. Then, Nekemte specialized hospital and Nekemte Health Center wrote a permission letter to respective PMTCT clinics. As the study was conducted through a review of medical records, patient confidentiality was kept. Moreover, no personal identifier was used on the data collection checklist.

## Results

### Socio-demographic characteristics of HIV positive pregnant and lactating women

From January 2015 –December 1, 2019, 516 pregnant and lactating women were started ART at Nekemte specialized hospital and Nekemte health center. Of the total women who started ART, 19 patients’ cards were not available. Three hundred ninety-three (393) patient cards were reviewed and 13 patient cards were excluded due to incompleteness of the data (outcome variable is not recorded). Finally, 380 patient cards with complete data were included in the final analysis. The mean age of the women was 26.6 years with SD of ± 4.4. One hundred seven (28.2%) of the women were ≤24 years and 174 (45.8%) belong to the age group 25–29 years. Greater than half (60%) of the women reside in urban and One hundred eighty-two (47.9%) of women were protestant religion followers. One hundred eighty two (47.9%) of women were protestant religion followers and 38.7% were orthodox religion followers. Regarding the employment status of participants, 175 (45.5%) was housewife (**Table** **1**).

**Table 1:** Baseline socio-demographic characteristics of HIV positive pregnant and lactating women at Nekemte public health facilities, western Ethiopia, 2015–2019.

Variables	Category	Frequency	(%)
**Age**	≤24 years	107	28.2
	25–29 years	174	45.8
	≥30 years	99	26.1
**Residence**	Rural	155	40.8
	Urban	255	59.2
**Marital status**	Never married/single	63	16.6
	Married	250	65.8
	Widowed/divorced	67	17.6
**Educational status**	No formal education	96	25.2
	Primary	123	32.4
	Secondary and above	161	40.4
**Religion**	Protestant	182	47.9
	Orthodox	147	38.7
	Muslim	51	13.4
**Employment**	Housewife	173	45.5
	Gov’t employee	104	27.4
	Non gov’t employee	40	10.5
	Daily laborer	41	10.8
	Others	22	5.8

*others—students, not employed.

### Maternal characteristics of the HIV positive pregnant and lactating women

One hundred fifty-seven (41.3%) of women’s partner HIV status was negative. Regarding the previous history of HIV, two hundred twenty-one (67.0%) women had a previous history of HIV. On the last follow-up, 299 (90.6%) women were lactating and only 31 (9.4%) women were pregnant. Two hundred eighty-eight (87.3%) women were initiated lifelong ART at the pregnancy stage and two hundred sixteen (65.5%) of women had multiple pregnancies (**Table** **2**).

**Table 2:** Maternal characteristics of HIV positive pregnant and lactating women at Nekemte public health facilities, western Ethiopia, 2015–2019.

Variables	Categories	Frequency	Percent
**Partner HIV status**	Positive	130	34.2
	Negative	157	41.3
	Unknown	93	24.5
**Initiated ART on the date of HIV diagnosis**	No	320	84.2
	Yes	60	15.8
**Status of women on enrollment**	Pregnant	343	90.3
	Lactating	37	9.7
**Number of pregnancy**	Single	131	34.5
	Multiple	149	65.5
**Previous History of HIV**	Yes	86	22.6
	No	294	77.4

### Baseline clinical, laboratory and follow up characteristics

At baseline, 312 (82.1) of women were in WHO stage I and 300 (79%) of women CD4 level was greater than 350 cells/μL. The majority (94.2%) of the women baseline functional status was working and a half (50.5%) received Cotrimoxazole preventive therapy (CPT) at baseline. The majority, 92.9% of women were negative for TB screening and three hundred twenty-five (85.5%) of the women’s had no previous history of comorbidity. Three hundred forty-five (90.8%) of the women had no baseline opportunistic infections, and 344 (90.5%) of women had no OI’s at a recent history (**Table** **3**).

**Table 3:** Baseline clinical and laboratory characteristics of HIV positive pregnant and lactating women at Nekemte public health facilities, western Ethiopia 2015–2019.

Variables	Category	Frequency	Percent
**WHO stage**	Stage I	312	82.1
	Stage II	45	11.8
	Stage III	22	5.8
	Stage IV	1	0.3
**CD4 level**	≤350 cells/μL	80	21
	≥351 cells/μL	300	79
**Hemoglobin**	≤12.0 g/dl	176	46.3
	≥12.1 g/dl	204	53.7
**Baseline functional status**	Working	358	94.2
	Ambulatory/bedridden	22	5.8
**Baseline OIs**	No	345	90.8
	Yes	35	9.2
**Baseline CPT**	No	188	49.5
	Yes	192	50.5
**Baseline TB screening**	Positive	27	7.1
	Negative	353	92.9
**Baseline comorbidity**	No	325	85.5
	Yes	55	14.5
**Baseline regimen**	TDF/3TC/EFV	276	72.6
	AZT/3TC/NVP	58	15.3
	AZT/3TC/EFV	14	3.7
	TDF/3TC/NVP	32	8.4
**Recent adherence**	Good	339	89.2
	Fair	28	7.4
	Poor	13	3.4
**Recent Drug side effect**	No	286	75.3
	Yes	94	24.7
**Recent OI’s**	No	344	90.5
	Yes	36	9.5

OI’s-Opportunistic Infections; CPT; Cotrimoxazole Preventive Therapy; TDF: Tenofofir; 3TC: Lamivudine; EFV: Efavirenz; NVP: Nevirapine; AZT: Zidovudine.

### Disclosure status of HIV positive pregnant and lactating women

**Fig 1:**
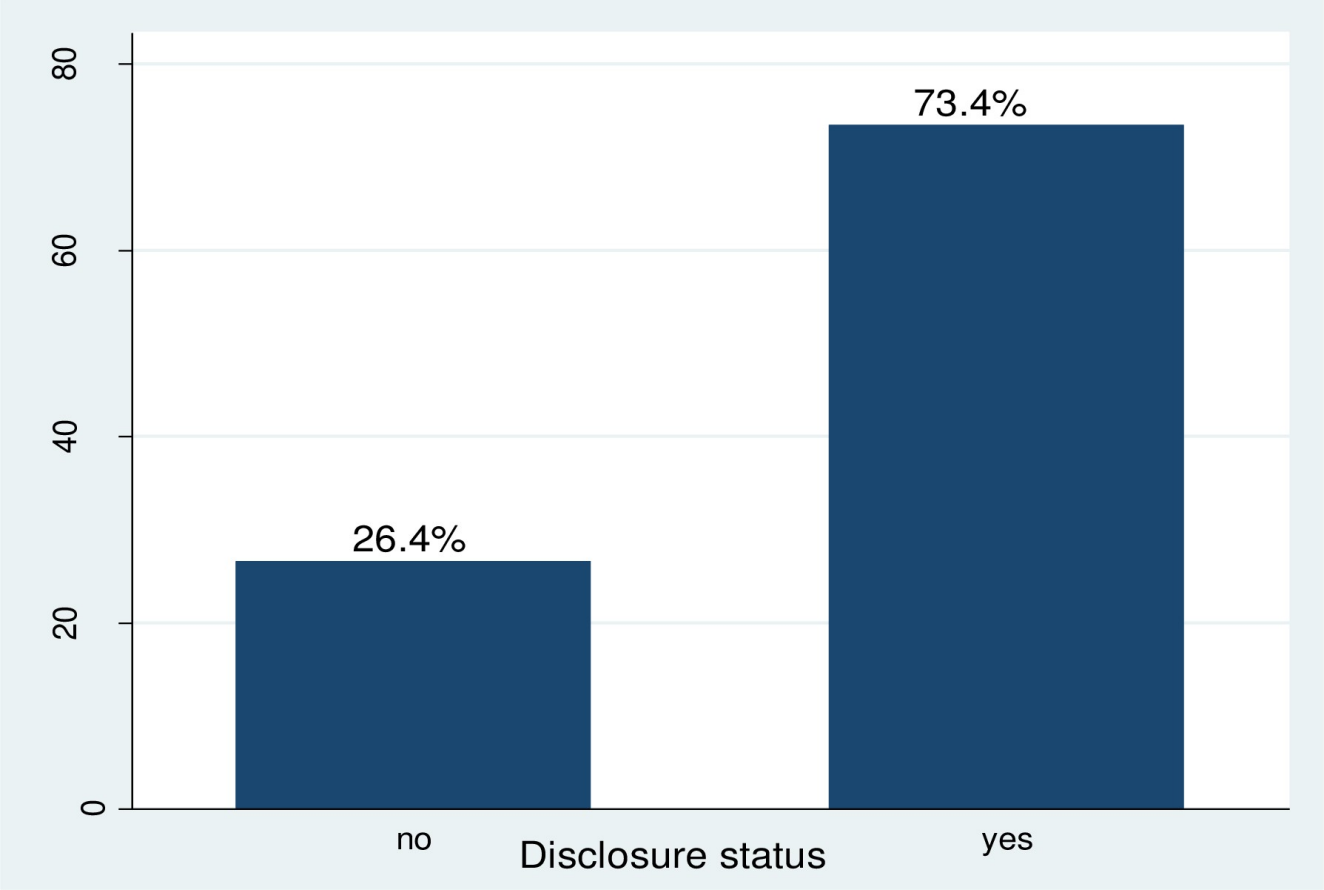
HIV status disclosure among HIV positive pregnant and lactating women at Nekemte public health facilities, western Ethiopia, 2015–2019.

A total of 380 women have participated in the study. Two hundred seventy-six (73.4%) indicated that they have disclosed their result to at least one individual (partners, relatives, brother/.sister and spouse). Nearly third-fourth of the respondents disclosed HIV status to their husband (71.2%), followed by children (5.78%), brother/ sister (7.55%), and other relatives (4.3%) (**Fig 1**).

### Factors associated with HIV disclosure status

To select variables for multivariable logistic regression, bivariable regression at p-value ≤ 0.25 was used. Then full multivariable analysis was conducted by using a forward stepwise selection process including all the potential risk factors that had a P-value of ≤ 0.25 in the bivariable analysis. Finally, five variables: residence, educational status, marital status, partner HIV status, and number of pregnancy on entry to the program were found to be significantly associated with disclosure status (**Table** **4**).

**Table 4:** Multivariable analysis of disclosure status and associated factors among HIV positive pregnant and lactating women at Nekemte public health facilities, western Ethiopia, 2015–2019.

Variables	Category	Disclosure status		COR	AOR	P-value
		Disclosed	Not disclosed			
**Residence**	Rural	99	56	1	1	
	Urban	180	45	2.26 (1.42, 3.59)	1.83 (1.04, 3.20)	0.034*
**Age**	≤24 years	50	57	1	1	
	25–29 years	146	28	5.94 (3.41, 10.35)	1.69 (0.79, 3.62)	0.176
	≥30 years	83	16	5.91 (3.08, 11.39)	1.13 (0.45, 3.29)	0.680
**Educational status**	No education	65	31	1	1	
	Primary	79	44	0.85 (0.48, 1.50)	1.18 (0.59, 2.34)	0.635
	Sec and above	135	26	2.47 (1.35, 4.50)	2.69 (1.31, 5.51)	0.007*
**Marital status**	Never married	29	34	1	1	
	Married	202	48	4.93 (2.74, 8.87)	4.16 (1.87, 9.24)	0.001*
	Widowed & divorced	48	19	2.96 (1.43, 6.12)	1.54 (0.61, 3.83)	0.353
**Number of pregnancy**	Single	63	68	1	1	
	Multiple	216	33	7.06 (4.27, 11.66)	4.94 (2.29, 10.66)	0.000*
**Partner HIV status**	Negative	108	22	1	1	
	Positive	108	49	2.12 (1.26, 3.93)	2.35 (1.17, 4.70)	0.015*
	Unknown	63	30	0.95 (.54, 1.652)	2.18 (1.01, 4.68)	0.045*
**Status of women on initiation of ART**	Pregnant	246	88	1	1	
	Lactating	33	13	0.90 (0.45, 1.80)	1.19 (0.52, 2.73)	0.674
**Hemoglobin level**	≤12.0 g/dl	128	48	1	1	
	≥12.1 g/dl	151	53	1.06 (0.67, 1.68)	1.12 (.65, 1.95)	0.667

AOR: Adjusted Odds Ratio; COR: Crude Odds Ratio

*statistically significant at p<0.05.

Women who are residing in urban were 1.83 times more likely to disclose their HIV status than those who are living in rural (OR = 1.83, 95% CI: 1.04, 3.20). The odds of disclosing their HIV status were 4.16 times higher among women who were married as compared to women were not married (OR = 4.16, 95% CI: 1.87, 9.24). Women who had attended secondary and above educational status were 2.69 times more likely to disclose HIV status as compared to those who had not attended formal education (OR = 2.35, 95% CI: 1.31, 5.51). The odds of disclosing HIV status were 2.35 times higher among women who had HIV positive partner as compared to those who have a negative HIV status partner (OR = 2.35, 95%CI: 1.17, 4.70. Multiparous women were 4.94 times more likely to disclose HIV status than women who have single pregnancy during entry to PMTCT program (OR = 4.94, 95% CI: 2.29, 10.66).

## Discussion

Disclosure of HIV status is among the contributing factors of good ART adherence for people receiving ART treatment, which indirectly improves the life expectancy of people living with human immunodeficiency virus (PLWH). It is an entry point for successful ART treatment. The current study pointed out that among pregnant and lactating women 73.4% disclose their serostatus to at least one person in the study area. This is lower compared to the studies done in Uganda that ranges from 83.7% to 85.4% [6, 15] and Kisarawe district hospital of Tanzania (98%) [16]. The discrepancy might be the information difference about the importance of HIV status disclosure among the study population. Similarly, it is lower than the study done in Jimma town (86.1%) [17], Northwest Ethiopia (89.7%) [12], and Addis Ababa (80.6%) [18]. The possible explanation for discrepancy could be variation in sample size or in awareness level among women. In contrary, this study finding is slightly higher than the study done in South Africa (71%) [19], and Kilimanjaro region of Tanzania(66%) [20]. This might be due to fear of intimate partner violence following disclosure in the previous studies [21].

However in the present study, the magnitude of pregnant women HIV status disclosure to their husband was 71.2%. This is consistent to the study finding from six health facilities located in the three districts of Central Uganda (73.3%) [6], and Central African Republic, Bangui (70.3%) [22]. In another way, it is higher than the study finding from Addis Ababa (51.7%) [18], Mbarara Regional Referral Hospital of Uganda(57%) [15], Mekelle Hospital (63.8%) [13], South Africa (61%) [19], and Kisarawe district hospital of Tanzania (56.3%) [16]. The discrepancy might be due to the time the previous studies were carried out; as currently the awareness level of the population is increasing.

Regarding the factors influencing disclosure status, the study revealed that the likelihood of HIV status disclosure was 1.83 times higher among women from urban dwellers than their counter parts. This is consistent with the study finding from Mbarara Regional Referral Hospital of Uganda [15], and Northwest Ethiopia [12]. The possible explanation could be women from rural area lack access to information about the importance of HIV status disclosure. They believe disclosure might results in stigma, social discrimination and isolation. In addition, the probability of HIV status disclosure was 4.16 times higher among married women as compared to unmarried women. This is supported with existing evidence from Tanzania and Southwest Ethiopia [20, 23]. In addition, a study from Central African Republic also found consistent finding that couples living separately was barrier for HIV status disclosure [22]. This might be due to the fact that married women feel responsible for their husband and children to be protected from HIV and hence they disclose their status to ensure that their families get protected. On the contrary, unmarried women are negligent about the transmission of the virus and more worried about their future life like marrying, childbearing and social relations; so that they kept silent about their HIV status.

Moreover, the current study found that the likelihood of HIV status disclosure was 2.35 times higher among women who have HIV seropositive husband compared to those who have husband of negative HIV status. This finding is in line with the study findings from South America French Guiana, Addis Ababa, and Jimma University specialized hospital [18, 23, 24]. The explanation for this might be the previous experience they had on the importance of disclosure or their frank discussion on the HIV status of her husband in the recent time. In addition, this could be explained as women who know her husband was HIV seropositive never fear abandonment from her partner and could easily disclose her HIV status.

Furthermore, the present study found out that a higher likelihood of HIV status disclosure among women who achieved secondary and above educational levels than those who have no formal education. A consistent finding was observed from Rwanda and Uganda [15, 25]. This depicts that education plays a key role in fostering awareness of women on the importance of HIV status disclosure which leads to a frank discussion with partner on medical care and treatment, protecting the family and improving their life expectancy. Though we couldn’t found evidence supporting this finding, the probability of HIV status disclosure was 4.94 times higher among multiparous women than uniparous women. The possible explanation might be multiparous women might have more exposure to counseling about disclosure during their antenatal care and Postnatal care service than uniparous women.

This study is subjected to some limitations. First, it is a retrospective review of records, which further limited due to unregistered outcome and limited information regarding potential variables which would be associated with disclosure status. Second, the true prevalence of disclosure might be underestimated and/or overestimated due to the incomplete documentation of data.

## Conclusion

HIV status disclosure among pregnant and lactating women in the study area was sub-optimal. The study found residence, marital status, HIV status of the husband, parity, and educational status as independent determinants of HIV status disclosure. Addressing women from the countryside by providing information on the importance of HIV status disclosure to ensure they get the necessary support from family members and health care system. Moreover, empowering women through education and enhancing active male involvement in HIV treatment and control programs should get due attention. For researchers, we will recommend conducting further prospective cohort and qualitative study to clearly identify factors that would be associated with disclosure status.

## Supporting information

S1 File STATA dataset. (DTA)

## Abbreviations

AOR: Adjusted Odds Ratio
CI: Confidence Interval
COR: Crude Odds Ratio
CPT: Cotrimoxazole Preventive Therapy
HIV: Human Immune Virus
OI’s: Opportunistic Infections
OR: Odd Ratio
PLWH: People living with HIV
PMTCT: Prevention of Mother to Child Transmission
SSA: Sab Saharan Africa
WHO: World Health Organization
